# Predicting direct and indirect breeding values for survival time in laying hens using repeated measures

**DOI:** 10.1186/s12711-015-0152-2

**Published:** 2015-09-28

**Authors:** Tessa Brinker, Esther D. Ellen, Roel F. Veerkamp, Piter Bijma

**Affiliations:** Animal Breeding and Genomics Centre, Wageningen UR, P.O. Box 338, 6700 AH Wageningen, The Netherlands

## Abstract

**Background:**

Minimizing bird losses is important in the commercial layer industry. Selection against mortality is challenging because heritability is low, censoring is high, and individual survival depends on social interactions among cage members. With cannibalism, mortality depends not only on an individual’s own genes (direct genetic effects; DGE) but also on genes of its cage mates (indirect genetic effects; IGE). To date, studies using DGE–IGE models have focussed on survival time but their shortcomings are that censored records were considered as exact lengths of life and models assumed that IGE were continuously expressed by all cage members even after death. However, since dead animals no longer express IGE, IGE should ideally be time-dependent in the model. Neglecting censoring and timing of IGE expression may reduce accuracy of estimated breeding values (EBV). Thus, our aim was to improve prediction of breeding values for survival time in layers that present cannibalism.

**Methods:**

We considered four DGE–IGE models to predict survival time in layers. One model was an analysis of survival time and the three others treated survival in consecutive months as a repeated binomial trait (repeated measures models). We also tested whether EBV were improved by including timing of IGE expression in the analyses. Approximate EBV accuracies were calculated by cross-validation. The models were fitted to survival data on two purebred White Leghorn layer lines W1 and WB, each having monthly survival records over 13 months.

**Results:**

Including the timing of IGE expression in the DGE–IGE model reduced EBV accuracy compared to analysing survival time. EBV accuracy was higher when repeated measures models were used. However, there was no universal best model. Using repeated measures instead of analysing survival time increased EBV accuracy by 10 to 21 and 2 to 12 % for W1 and WB, respectively. We showed how EBV and variance components estimated with repeated measures models can be translated into survival time.

**Conclusions:**

Our results suggest that prediction of breeding values for survival time in laying hens can be improved using repeated measures models. This is an important result since more accurate EBV contribute to higher rates of genetic gain.

**Electronic supplementary material:**

The online version of this article (doi:10.1186/s12711-015-0152-2) contains supplementary material, which is available to authorized users.

## Background

Minimizing bird losses in the commercial layer industry is important, both from welfare and economic points of view. Thus, selection against mortality has been of interest to researchers [1–3] but has not always been effective [4]. Genetic improvement of mortality in poultry breeding is challenging for several reasons. In addition to having a low heritability, one of the main complications is that the time until death is often not observed because most laying hens are still alive at the end of the recording period [5, 6]. Hence, only a lower bound of the true survival time is known for most hens, which is referred to as censoring [5]. Excluding censored records from analyses or considering the lower bound as the actual record is expected to reduce the accuracy of estimated breeding values (EBV).

The fact that commercial laying hens live in groups complicates selection for lower mortality even more. Group housing allows social interactions between group members, such that survival time in laying hens might be adversely affected by harmful social behaviours such as feather pecking [7, 8]. In these cases, survival time depends on both the genes of the potential victim (known as the direct genetic effect; DGE) and on the genes of its cage mates (known as the indirect genetic effect; IGE) [2, 9–13]. In other words, the environment that individuals experience contains a heritable component (IGE), expressed by the cage mates. Such IGE can affect response to selection considerably and neglecting IGE when selecting for lower mortality can even result in a negative response to selection [1, 14].

Ellen et al. [15] and Peeters et al. [16] investigated the contribution of IGE to heritable variation in survival time of laying hens. These two studies used a DGE–IGE linear mixed model to estimate genetic parameters. Shortcomings of this model are that censored records were considered as exact lengths of life and it assumed that IGE were continuously expressed by all individuals in a cage, irrespective of whether they were alive or dead. The latter assumption is invalid because cage composition changes over time due to death of animals, as dead animals no longer express IGE on their cage mates. Thus, to increase the accuracy of estimates of DGE and IGE for survival time, methods that can cope with censoring and timing of IGE expression should be explored.

Ellen et al. [17] investigated opportunities of survival analysis models with DGE and IGE to account for censoring. Survival analysis models exploit information on both censored and uncensored records properly by accounting for the non-linearity of survival time data and can also include time-dependent effects [18, 19]. Ellen et al. [17] used a two-step approach that combined survival analysis and a DGE–IGE linear mixed animal model for survival time because it was not possible to estimate the variance of correlated genetic effects with existing survival analysis software. However, Ellen et al. [17] showed that the accuracy of EBV was not improved with the two-step approach compared to the DGE–IGE linear mixed animal model for data in which all surviving animals were censored at the same time.

Several other statistical techniques for analysing survival data have been proposed that can consider censoring and time-dependent effects, including repeated measures models [20–24]. In repeated measures models, a survival indicator is used to circumvent censoring. Survival is measured as a binomial trait (0/1), which indicates that an individual is dead (0) or alive (1) at specific time points [24], or that an individual has survived or not during a specific time period [22]. In the first case, survival has no missing records, whereas in the second case, records for time periods after death of an individual are set to missing. The trait definition differs in each approach since the method of Jamrozik et al. [24] approximates the survival function of the proportional hazard model, whereas the method of Veerkamp et al. [22] approximates the hazard function of the proportional hazard model. Modelling the hazard function enables to estimate covariances between independent time intervals whereas modelling the survival function enables to estimate covariances between cumulative averages of time intervals [25].

In a study on survival in dairy cows, Veerkamp et al. [22] found that the repeated measures model was robust to censoring since the correlations between EBV from uncensored and randomly censored data were high. Ødegård et al. [26] confirmed this finding in a comparative analysis of different models for survival data in Atlantic salmon. They showed that the repeated measures model had a greater predictive ability than survival analysis [26]. Thus, repeated measures models appear to be an appropriate tool for analysing survival data since they can account for censoring and timing of IGE expression. However, to date, the potential of repeated measures models for estimating DGE and IGE on survival time has not been investigated.

The aim of this study was to improve prediction of breeding values for direct and indirect effects on survival time in two purebred White Leghorn layer lines in which the level of mortality was high due to cannibalism [15]. For this purpose, we compared EBV for survival (0/1) from repeated measures models to EBV for survival time from a linear mixed model. The predictive ability of EBV from both models was assessed by cross-validation.

## Methods

### Populations and pedigree

Data were collected under the control of the Institut de Sélection Animale B.V. (ISA), the layer breeding division of Hendrix Genetics. Hendrix Genetics complies with the Dutch law on animal welfare. ISA provided data on two purebred White Leghorn layer lines, denoted W1 and WB [15].

For each line, matings between sires and dams were randomly assigned and occurred in two batches with a 6-month interval. For each batch, each sire (36 for line W1 and 35 for line WB) was mated to approximately eight dams, resulting in an average of 12.3 female offspring per dam. Each batch was partitioned into four age groups that differed in age by 2 weeks. Laying hens had intact beaks.

### Housing

Laying hens of the same age were randomly allocated to four-bird battery cages approximately 17 weeks after hatching. Each batch was transported to a different laying house, coded as 1 and 2. The laying houses had eight double rows of cages and each row comprised three levels (top, middle, and bottom). Hens in laying house 2 were not placed on the top level. A standard commercial layer diet and water were provided ad libitum in the front and back parts of each cage, respectively. Light intensity was stronger in laying house 2 than in laying house 1 [15]. Further details are in Ellen et al. [15].

### Data

**Fig. 1:**
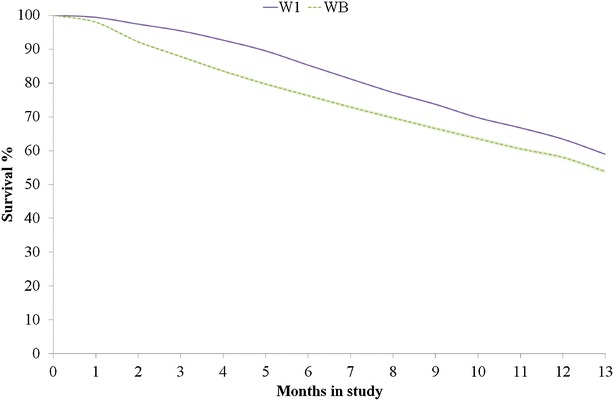
Percentage of survival of layer chickens for lines W1 and WB throughout the experiment (max = 13 months)

**Fig. 2:**
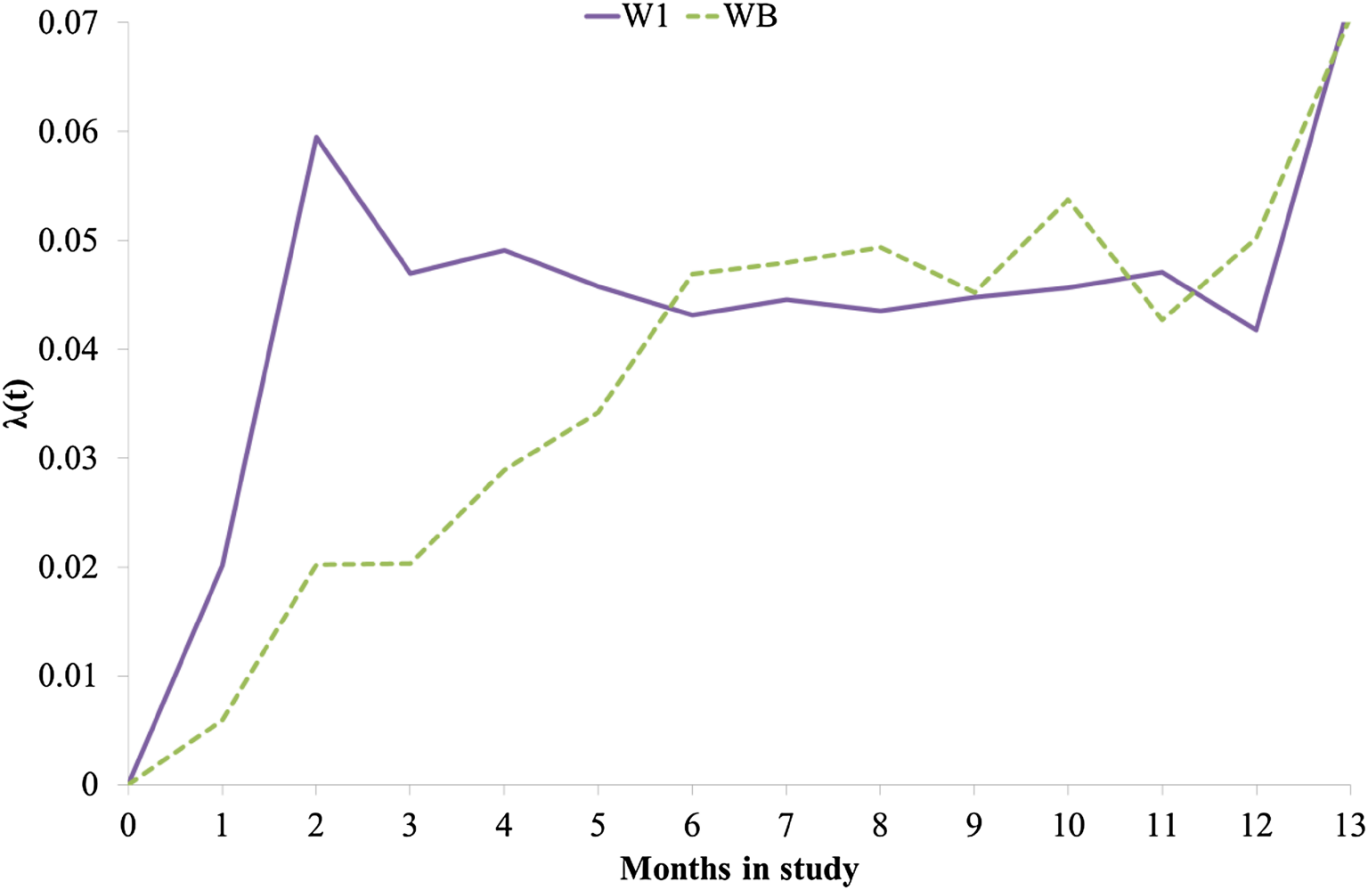
Hazard function λ(t) of layer chickens for lines W1 and WB throughout the experiment (max = 13 months)

Dead hens were removed daily. After death, wing band number, cage number, date of death, and cause of death were recorded. The latter was done subjectively by the employees of ISA without dissection. The study was terminated when hens were on average 75 weeks old. In total, 59 % W1 and 54 % WB laying hens survived (Fig. 1). Survival rates of W1 and WB hens differed most during the first 4 months of the experiment (Fig. 2). Most hens died because of cannibalism; only 37 W1 and 15 WB hens died for other reasons, e.g. some hens were killed by mink. Observations on hens that died for other reasons were removed from the dataset because the objective was to investigate death from cannibalism. However, the identification numbers were retained in their cage mates’ observations for IGE modelling.

Survival time was defined as the number of days from entry in the laying house to death. Since each batch consisted of four age groups that differed in age by 2 weeks, the maximum number of survival days differed between age groups. Thus, the maximum number of survival days was cut off at 416 days, which is the maximum survival time of the youngest age group, which means that all hens that were still alive at 416 days of age had censored records on survival time. In the statistical analysis of survival time, those hens were given a value of 416 days. In total, records on 6276 and 6916 hens were used for statistical analysis of survival time for lines W1 and WB, respectively.

To define survival, the laying period was divided into 13 months. For each month, survival was coded as 1 if the laying hen was alive at the end of that month, and as 0 if not. Thus, a survival record (0/1) was available for each month. This resulted in a total of 81,588 and 89,908 monthly records for lines W1 and WB, respectively.

### Statistical models

Four statistical models were compared: a linear mixed model for survival time, two linear mixed models for survival (0/1), and a generalized linear mixed model for survival (0/1). Five generations of pedigree were included in all genetic analyses. All models were implemented using ASReml [27].

#### Survival time model STM

DGE and IGE for survival time were estimated using a survival time model (STM) [11, 12] with the following linear mixed model:

1 $$y_{ijk} = fixed + A_{{D_{i} }} + \mathop \sum \limits_{j \ne i}^{n - 1} A_{{I_{j} }} + cage_{k} + e_{ijk} ,$$

where *y*_*ijk*_ is the observed survival time (days) for individual *i*, with cage mates *j*, in cage *k*, the *fixed* term is the fixed effect of the combination of laying house-row-level, $A_{{D_{i} }}$ is the random DGE of individual *i*, $\sum\nolimits_{j \ne i}^{n - 1} {A_{{I_{j} }} }$ is the sum of the *n* − 1 random IGE of the cage mates *j,* with *n* denoting cage size at the start of the experiment (*n* = 4), *cage*_*k*_ is the random cage effect, and *e*_*ijkl*_ is the residual. Individuals that were still alive at the end of the recording period were assigned a survival time record of 416 days. Genetic effects were assumed to follow a normal distribution $\sim N(0,{\mathbf{C}} \otimes {\mathbf{A}})$, with $${\mathbf{C}} = \left[ {\begin{array}{*{20}c} {\sigma_{{A_{D} }}^{2} } & {\sigma_{{A_{DI} }} } \\ {\sigma_{{A_{DI} }} } & {\sigma_{{A_{I} }}^{2} } \\ \end{array} } \right]$$, the Kronecker product of matrices $\otimes$, a relationship matrix **A**, direct genetic variance $\sigma_{{A_{D} }}^{2}$, indirect genetic variance $\sigma_{{A_{I} }}^{2}$, and direct–indirect genetic covariance $\sigma_{{A_{DI} }}$.

Residuals of cage members may be correlated because of non-heritable indirect effects, with: $\rho = \left( {2\sigma_{{E_{DI} }} + \left( {n - 2} \right)\sigma_{{E_{I} }}^{2} } \right)/\sigma_{e}^{2}$ [12]. In cases where cage members are ‘similar’, i.e. when $\rho$ is positive, a random cage effect can be fitted instead of fitting correlated residuals, with $\sigma_{cage}^{2} = 2\sigma_{{E_{DI} }} + \left( {n - 2} \right)\sigma_{{E_{I} }}^{2} = \rho \sigma_{e}^{2}$. Based on a previous study [15], the correlation was estimated to be positive, and a random cage effect was therefore fitted in this study. The cage effect cannot be fitted as a fixed effect because the indirect genetic variance is not statistically identifiable when a fixed cage effect is included [28].

#### Repeated measures model RMM.t

Monthly survival (0/1) was analysed using a repeated measures model that included random DGE and IGE regressions on time (hence RMM.t) based on a sire-dam model. No genetic effects for intercept were fitted, because there is no phenotypic variation at the start of the experiment (*t* = 0), since all hens were alive at time 0. After that, phenotypic variance in survival increases over time until it reaches a maximum at 50 % mortality, and then declines again. In this experiment, mortality was less than 50 %, so phenotypic variance only increased over time. This increase in variance over time is consistent with a model with random regressions on time, for which the variance is proportional to the square of time. The model with random regressions on time was:

2 $$y_{ijklm} = fixed + A_{{sd_{Di} }} \cdot {\text{t}}_{m} + \mathop \sum \limits_{j \ne i}^{n - 1} A_{{sd_{Ij} }} \cdot t_{m} + cage_{km} + cage_{k} \cdot t_{m} + PE_{l} \cdot t_{m} + e_{ijklm} ,$$

where *y*_*ijklm*_ is the observed survival (0/1) for individual *l,* offspring of sire-dam combination *i*, with cage mates from sire-dam combination *j,* in cage *k*, at time *t*_*m*_ measured in months since entry of the experiment, the *fixed* term is the fixed interaction effect of laying house-row-level, which was fitted with a sixth-order polynomial of time, $A_{{sd_{Di} }}$ is the DGE of the sire-dam combination as a function of time, $\sum\nolimits_{j \ne i}^{n - 1} {A_{{sd_{Ij} }} }$ is the sum of the *n* − 1 IGE of the sire-dam combination of the cage mates as a function of time, *cage*_*km*_ is the random effect of cage *k* at time *m*, *cage*_*k*_ is the random effect of cage *k* as a function of time, *PE*_*l*_ is the random permanent environmental effect of individual *l* as a function of time, *t*_*m*_ is the time, and e_*ijklm*_ is the residual. A separate residual variance was estimated for each month. The DGE and IGE were allowed to be correlated. Sire-dam effects were assumed to follow a normal distribution $\sim N(0,{\mathbf{C}} \otimes {\mathbf{A}})$, with $${\mathbf{C}} = \left[ {\begin{array}{*{20}c} {\sigma_{{A_{{D_{sd} }} }}^{2} } & {\sigma_{{A_{{D_{sd} I_{sd} }} }} } \\ {\sigma_{{A_{{D_{sd} I_{sd} }} }} } & {\sigma_{{A_{{I_{sd} }} }}^{2} } \\ \end{array} } \right]$$, direct genetic sire-dam variance $\sigma_{{A_{{D_{sd} }} }}^{2}$, indirect genetic sire-dam variance $\sigma_{{A_{{I_{sd} }} }}^{2}$, and direct–indirect genetic sire-dam covariance $\sigma_{{A_{{D_{sd} I_{sd} }} }}$.

This model contains two random cage effects: one is a random cage effect at each time, *cage*_*km*_, and the other, $cage_{k} \cdot t_{m}$, is a random regression on time. The *cage*_*km*_ effect accounts for covariances among cage members at specific time points. A single variance was estimated for this effect. The $cage_{k} \cdot t_{m}$ term accounts for covariances between records on the same cage at different time points, and for increasing variance over time. Both random cage effects were very significant and when excluding one or the other, the variance explained by cage was not fully covered.

#### Repeated measures model RMM.p

The regressions on time in the RMM.t model (Eq. 2) imply that variances are a quadratic function of time; e.g. $var\left( {a \cdot t} \right) = t^{2} var(a)$, where *a* is the genetic effect and *t* is time. However, the true variance for binomial traits equals $p\left( {1 - p} \right)$, where *p* is the probability for an individual to survive until time *t*. In other words, *p* is the mean survival at time *t*. A quadratic function of time does not fit a $p\left( {1 - p} \right)$ function well because the slope of $t^{2}$ increases with *t*, whereas the slope of $p\left( {1 - p} \right)$ decreases with *p*. To better fit the variance, the regression on time was replaced by a regression on $\sqrt {p(1 - p)}$, so that fitted variances were proportional to $p\left( {1 - p} \right)$; e.g. $var\left( {a \cdot \sqrt {p\left( {1 - p} \right)} } \right) = p\left( {1 - p} \right) \cdot var\left( a \right)$. In other words, monthly survival (0/1) was analysed using a repeated measures model including DGE and IGE regressions on a function of mean survival (RMM.p), rather than on time. The model was:

3 $$y_{ijklm} = fixed + A_{{sd_{Di} }} \cdot x_{m} + \mathop \sum \limits_{j \ne i}^{n - 1} A_{{sd_{Ij} }} \cdot x_{m} + cage_{km} + cage_{k} \cdot x_{m} + PE_{l} \cdot x_{m} + e_{ijklm} ,$$

where $x_{m} = \sqrt {p_{m} (1 - p_{m} )}$, *p*_*m*_ denoting the mean survival at time *m*. For each time *m*, the *p*_*m*_ was calculated separately for each fixed effects class. The other terms are the same as for Eq. 2.

#### Generalized linear mixed model GLMM

To account for the binomial distribution of monthly survival, DGE and IGE for survival were estimated using a generalized linear mixed model (GLMM) with a logit link function. The GLMM was:

4 $$\eta (E(y_{ijklm} )) = fixed + A_{{sd_{Di} }} + \mathop \sum \limits_{j \ne i}^{n - 1} A_{{sd_{Ij} }} + cage_{km} + PE_{l} ,$$

where $\eta (_{ } )$ is the logit link function that links the probability *p* of surviving to the linear predictor, $E(y_{ijklm} )$ is the probability of survival for individual *l*, offspring of sire-dam combination *i*, with cage mates from sire-dam combination *j*, in cage *k*, at time *m*. The other terms are the same as for Eq. 2.

In contrast to the survival models, the GLMM only includes a genetic intercept rather than a regression on time. This is because the non-linear link function takes the change in variance over time into account. Hence, at the beginning of the recording period, the variance of survival probabilities can be (near) zero even when $var[\eta (E(y_{ijklm} ))]$ is substantially greater than 0.

### Time-dependent IGE

The data structure of the survival models (RMM.t, RMM.p, and GLMM) allows inclusion of time-dependent random effects. The composition of the cage changes over time because animals die. Thus, we tested if the prediction of breeding values could be improved by including timing of IGE expression in the model. For this purpose, the cage mates that were alive at the beginning of each month were indicated for each monthly survival observation. Hence, in models RMM.t, RMM.p and GLMM, the $\sum\nolimits_{j \ne i}^{n - 1} {A_{{sd_{Ij} }} }$ was time-dependent, and included only sires-dam combinations of cage mates that were still alive at the beginning of the month. Thus, the sires and dams of cage mates that were no longer alive were set to missing. For time periods after death of the focal individual, the cage composition was kept identical to that at the time of death because these observations were no longer impacted by changes in cage composition.

### Cross-validation

The quality of EBV from each model was assessed by cross-validation, which is a technique of model validation where the correlation between predicted and observed phenotypes serves as a quality measure [29]. With this procedure, known phenotypes are set to missing, their values are predicted, and finally the predicted values are compared to the observed values. In this study, five mutually exclusive subsets were created, where survival phenotypes for approximately 20 % of the cages were removed. Those cages were selected at random. These subsets were used to predict the phenotypes of the individuals that belonged to the 20 % cages that were removed. All fixed effect classes were present for each subset. A bivariate analysis in ASReml [27] of the ranks of observed and predicted phenotypes, with a fixed effect for each subset (from 1 to 5), was used to calculate the Spearman rank correlation and corresponding standard error across the five validation sets [27]. We used rank correlations because survival phenotypes were unknown for the censored individuals (see below). The following sections describe how observed and predicted phenotypes were obtained.

#### Observed and predicted phenotypes

For the individuals that were not censored, observed phenotypes were the observed survival days. First, these were adjusted for fixed effects using a linear model with fixed effects only; **y** = **Xb** + **e**, with **y** being a vector of observed survival times, **X** being an incidence matrix linking survival time observations to fixed interaction effects of laying house-row-level, **b** being a vector of the fixed effects, and **e** is the residual term. Hence, the residual of this model represents the adjusted phenotypes. For the censored individuals, the observed phenotype is unknown but these observations contain important information because they correspond to hens with the largest number of survival days. To allow calculation of the rank correlation between predicted and observed phenotypes, we followed the approach presented in [17], which assumes that censored individuals died in random order after surviving up to 416 days. Under this assumption, censored individuals can be given the average rank of all censored individuals. For example, when 5 out of 10 individuals are censored, then the average rank of the censored individuals is (6 + 7 + 8 + 9 + 10)/5 = 8 and all censored individuals are given a rank of 8, while uncensored individuals were given their observed rank (after correction for fixed effects).

Predicted phenotypes were the rank of predicted survival times of individuals. Phenotypes were predicted by combining the estimated DGE ($\hat{A}_{{{\text{D}}_{i} }} )$ of the individual itself and the estimated IGE of its cage mates (*n* = 3) that were present at the start of the experiment $\left( {\sum {\hat{A}_{{{\text{I}}_{j} }} } } \right)$ (see Additional file 1 for details).

#### Approximate accuracy

Based on the method described in Ellen et al. [17], approximate accuracies of EBV were calculated for all models. These were approximations because accuracy refers to the ranks rather than to the phenotypic values and EBV themselves. If EBV underlying the predicted phenotypes were estimated with an accuracy of 1, the expected rank correlation would be:

5 $$\sqrt {r^{2} } = \sqrt {{{\left( {\sigma _{{A_{D} }}^{2} (n - 1)\sigma _{{A_{I} }}^{2} } \right)} \mathord{\left/ {\vphantom {{\left( {\sigma _{{A_{D} }}^{2} (n - 1)\sigma _{{A_{I} }}^{2} } \right)} {\sigma _{P}^{2} }}} \right. \kern-\nulldelimiterspace} {\sigma _{P}^{2} }},}$$

where the numerator is the genetic component of phenotypic variance. The approximate accuracy was calculated as:

6 $$\hat{r}_{IH} = corr{{\left[ {rank(P_{i} - \bar{P})_{i} , rank(\hat{P}_{i} )} \right]} \mathord{\left/ {\vphantom {{\left[ {rank(P_{i} - \bar{P})_{i} , rank(\hat{P}_{i} )} \right]} {\sqrt {r^{2} } }}} \right. \kern-0pt} {\sqrt {r^{2} } }},$$

where $P_{i} - \bar{P}$ represents the observed phenotype corrected for fixed effects, and $\hat{P}_{i}$ represents the predicted phenotype. Genetic parameter estimates from model STM were used to calculate $\sqrt {r^{2} }$ (see Table 1).

**Table 1:** Estimates of genetic parameters (±SE) for survival time using models STM, RMM.t, and RMM.t with time-dependent indirect genetic effects (RMM.t-td) for two layer lines W1 and WB

	W1			WB		
	STM	RMM.t	RMM.t-td	STM	RMM.t	RMM.t-td
$\sigma_{{A_{D} }}$	28 ± 3	28 ± 3	29 ± 3	41 ± 4	38 ± 4	41 ± 4
$\sigma_{{A_{I} }}$	10 ± 2	11 ± 2	33 ± 2	16 ± 3	12 ± 2	20 ± 1
$\sigma_{{A_{DI} }}$	57 ± 67	57 ± 64	255 ± 129	−158 ± 120	−111 ± 87	−311 ± 112
$\sigma_{TBV}$	45 ± 8	46 ± 7	109 ± 8	55 ± 9	46 ± 8	58 ± 7
$\sigma_{P}$	107 ± 1	107 ± 1	114 ± 1	135 ± 1	123 ± 1	128 ± 1
$T^{2}$	0.18 ± 0.06	0.19 ± 0.06	0.93 ± 0.11	0.16 ± 0.05	0.14 ± 0.05	0.21 ± 0.05
$r_{A}$	0.20 ± 0.22	0.19 ± 0.20	0.26 ± 0.13	−0.24 ± 0.18	−0.24 ± 0.19	−0.38 ± 0.13

Estimates of genetic parameters are provided for survival time in days for both W1 and WB lines. $\sigma_{{A_{D} }}$, $\sigma_{{A_{I} }}$, and $\sigma_{{A_{DI} }}$ are the direct genetic standard deviation, indirect genetic standard deviation, and direct–indirect genetic covariance. $\sigma_{TBV}$ is the total genetic standard deviation, $\sigma_{P}$ is the phenotypic standard deviation, *T* ^2^ is the total heritable variance relative to the phenotypic variance, and $r_{A}$ is the genetic correlation between direct and indirect genetic effects. Additional file 1 describes the procedure to translate genetic parameters of RMM.t to survival days

### Genetic parameters

In addition to EBV, we were interested in estimating genetic parameters for survival time, which can, e.g., give an indication of the amount of genetic progress that can be made for a trait. To make genetic parameter estimates from the different models comparable, they were transformed to the survival time scale, as described in the following section for RMM.t. For RMM.p and GLMM, the translation of genetic parameters to the survival time scale involves tedious integrals and, thus, genetic parameters from these models are not presented here.

For STM, parameters for survival time follow directly from the estimates. In the presence of social interactions, where each individual interacts with *n* − 1 cage mates, the total heritable variance for response to selection is given by [12, 30]:

7 $$\sigma_{TBV}^{2} = \sigma_{{A_{D} }}^{2} + 2\left( {n - 1} \right)\sigma_{{A_{DS} }} + (n - 1)^{2} \sigma_{{A_{S} }}^{2} ,$$

phenotypic variance equals:

8 $$\sigma_{P }^{2} = \sigma_{{A_{D} }}^{2} + \left( {n - 1} \right)\sigma_{{A_{S} }}^{2} + \sigma_{cage}^{2} + \sigma_{e}^{2} ,$$

and the ratio of heritable variance and phenotypic variance is equal to:

9 $$T^{2} = \frac{{\sigma_{TBV}^{2} }}{{\sigma_{P}^{2} }}.$$

*T*^2^ is an analogy of heritability that expresses the total heritable variance that is available for response to selection relative to phenotypic variance [13, 30].

To obtain genetic parameters for survival time from the models for monthly survival (0/1), survival has to be translated into survival time. Survival time (*ST*) of an individual is the sum of its survival records (*S* = 0,1) for each day over time,

10 $$ST_{i} = c \cdot \mathop \sum \limits_{t = start}^{t = end} (S_{i} )_{t} ,$$

where *c* is a multiplication factor that translates monthly survival (0/1) into days; *c* = 30.4 days. With this relationship it is possible to translate the estimated genetic parameters for survival to the survival time scale (see Additional file 2). Hence, a survival model can be used to estimate genetic parameters and breeding values for survival time.

## Results and discussion

### Cross-validation

The rank correlations between observed and predicted phenotypes, $corr\left[ {rank(P_{i} - \bar{P})_{i} , rank(\hat{P}_{i} )} \right]$ and the corresponding approximate accuracy ($\hat{r}_{IH}$) of the four models (STM, RMM.t, RMM.p, and GLMM) are in Table 2. Rank correlations ranged from 0.135 to 0.162 for line W1, and from 0.170 to 0.190 for line WB. Corresponding $\hat{r}_{IH}$ ranged from 0.44 to 0.53 for line W1, and from 0.46 to 0.52 for line WB. Although rank correlations appear low, they are in fact within our range of expectations because if breeding values were predicted with an accuracy of 1, the rank correlation would be equal to the square root of the proportion of phenotypic variance explained by the genetic variance, i.e. $\sqrt {r^{2} }$ (Eq. 5) [17]. Using the genetic parameters from STM (Table 1), $\sqrt {r^{2} }$ is equal to 0.31 and 0.37 for lines W1 and WB, respectively. These values are the upper bounds of the rank correlations in Table 2.

**Table 2:** Rank correlations between observed and predicted phenotypes (±SE) and approximate accuracies for lines W1 and WB

Model	Time-dependent	Rank correlation				Approximate accuracy	
		W1	% Improvement^a^	WB	% Improvement^a^	W1	WB
STM	–	0.135 ± 0.012	–	0.170 ± 0.012	–	0.44	0.46
RMM.t	No	0.148 ± 0.012	+10	0.185 ± 0.012	+9	0.48	0.51
RMM.p	No	0.162 ± 0.012	+20	0.174 ± 0.012	+2	0.53	0.47
GLMM	No	0.150 ± 0.012	+11	0.190 ± 0.012	+12	0.49	0.52
RMM.t	Yes	0.063 ± 0.013	−53	0.134 ± 0.012	−21	0.20	0.37
RMM.p	Yes	0.049 ± 0.013	−64	0.124 ± 0.012	−27	0.16	0.34
GLMM	Yes	0.081 ± 0.013	−41	0.149 ± 0.012	−12	0.26	0.41

Rank correlations between observed and predicted phenotypes and approximate accuracies using STM, RMM.t, RMM.p, and GLMM are provided for lines W1 and WB. All models were analysed by either excluding (time-dependent = no) or including (time-dependent = yes) timing of IGE expression

^a^Compared to STM

#### Model comparison

Previous studies analysed survival time with an animal model [15–17]. Our objective was to investigate whether predictions from such models could be improved, and for this reason, STM was analysed with an animal model, while RMM.t, RMM.p, and GLMM were analysed with a sire-dam model. Sire-dam models were used because animal models may result in biased genetic parameter estimates because of the “extreme category problem” when analysing binomial/categorical data [31]. At the start of the experiment no variation exists, but as hens start to die the variation within classes of fixed effects starts to change. Moreover, for some classes of fixed effects, the variation is not apparent until later in the experiment. It is not an option to remove these fixed-effect classes because they contain important information on survival. Hence, we found sire-dam models more appropriate than animal models to predict breeding values and genetic parameters.

To justify these comparisons with STM, we investigated whether analysis of survival time using an animal model or a sire-dam model were comparable. Genetic parameters of survival time (see STM in Table 1) and rank correlations between observed and predicted phenotypes (see STM in Table 2) were the same for the animal model and the sire-dam model. In addition, correlations between EBV that were predicted for the cross-validation sets by the animal and sire-dam models were higher than 0.99 for both lines. Thus, sire and animal models for survival time gave very similar results.

Comparing EBV from RMM.t, RMM.p, and GLMM with EBV from STM showed that accuracies increased for all three models (Table 2), by up to 20 % for line W1, and 12 % for line WB. Model RMM.p resulted in the highest predictive ability for line W1 but led to little improvement over STM for line WB. Based on theory, RMM.p was expected to improve prediction of breeding values for both lines compared to RMM.t because RMM.p fits a variance that better agrees with the true binomial variance. However, the results using survival data on line WB were not in line with this expectation, which might be caused by differences in survival curves between both lines.

A comparison of GLMM with RMM.t indicated that the predictive ability of these two models was quite similar, although the GLMM resulted in 1 % (line W1) to 3 % (line WB) greater accuracies than RMM.t. The ranks of predicted phenotypes from GLMM and RMM.t were highly correlated: 0.97 for line W1 and 0.98 for line WB (Table 3), which means that there was almost no re-ranking by using GLMM or RMM.t to predict breeding values. This is in line with other studies that found no advantage of analysing binomial/categorical data with binomial/categorical models compared to using linear models [32, 33].

**Table 3:** Rank correlations between predicted phenotypes from models without timing of IGE expression (±SE)

Model	STM	RMM.t	RMM.p	GLMM
STM		0.876 ± 0.003	0.789 ± 0.005	0.883 ± 0.003
RMM.t	0.910 ± 0.002		0.878 ± 0.003	0.976 ± 0.001
RMM.p	0.795 ± 0.005	0.876 ± 0.003		0.881 ± 0.003
GLMM	0.926 ± 0.002	0.973 ± 0.001	0.874 ± 0.003	

Rank correlations between predicted phenotypes from STM, RMM.t, RMM.p, and GLMM excluding timing of IGE expression are provided for lines W1 (below the diagonal) and WB (above the diagonal)

Survival analysis is a commonly used method to deal with censoring and time-dependent effects [18, 19]. However, it is not possible to estimate the variance for correlated genetic effects with the current software for survival analysis. Veerkamp et al. [22] used a method that approximates survival analyses by analysing survival as dead (0), alive (1) or missing (for time periods after the death of an individual) with a repeated measures model regressed on time and an intercept. This is different from our study, in which we coded survival as either dead (0) or alive (1), without missing records, following Jamrozik et al. [24]. The method of Veerkamp et al. [22] has similarities with the hazard function used in survival analysis. For our data, we found that rank correlations obtained with the method of Veerkamp et al. [22] were lower than those obtained with RMM.t, RMM.p and GLMM and were similar or lower than those obtained with STM. When coding survival as dead (0), alive (1) or missing, rank correlations were equal to 0.132 and 0.157 for lines W1 and WB.

Ellen et al. [17] explored the potential of survival analysis by applying a two-step approach that combined survival analysis and STM. Similar to the Veerkamp et al. method [22], the Ellen et al. [17] method approximates survival analysis. Applying the method of Veerkamp et al. [22] to our data yielded results that were in line with those of Ellen et al. [17], who observed no improvement in rank correlations using the two-step approach compared to STM.

#### Time-dependent IGE

Including timing of IGE expression in the repeated measures models had a substantial negative effect on rank correlations; compared to the results obtained with STM, rank correlations decreased by 12 and 64 % for lines WB and W1, respectively (Table 2). Lipschutz-Powell et al. [34] performed a simulation study to investigate whether DGE–IGE models applied to infectious disease data could accommodate the dynamic nature of such data. The non-infected individuals in Lipschutz-Powell et al. [34] are equivalent to dead individuals in our study, since both traits do not express the IGE. Lipschutz-Powell et al. [34] found that accounting for timing of IGE expression in the DGE–IGE model inflated the variance for IGE, similar to what we observed (Table 1). They indicated that the problem probably arises from the fact that modification of the incidence matrices that link observations to IGE directly depends on the observations [34]. As a consequence, a considerable amount of the phenotypic variance will be explained by IGE and IGE variance will be overestimated. Other methods to incorporate timing of IGE expression need to be explored.

#### Censoring

Our results indicate that it is important to use methods that incorporate censoring when analysing survival time. With STM, only a lower bound of the true survival time is known for censored records, which results in reduced accuracy. In the repeated measures models, this problem was circumvented by using a survival indicator, i.e. dead (0) or alive (1) at a given time. In this case, accuracy of predictions of breeding values increased by up to 20 and 12 % for lines WB and W1, respectively, compared to accuracies from STM. In this study, censored records were obtained at the same time for all individuals, which is often the case in layer breeding programs. Censoring at various times during the laying period will result in different accuracies compared to what was presented in this study. We investigated the effect on predicted breeding values when 50 % of the censored individuals were censored half way during the laying period using STM and RMM.t. Compared to analyses where all hens were censored at the same time, rank correlations using STM decreased by 16 to 25 %, while rank correlations using RMM.t decreased by only 3 to 6 % (results not shown). Thus, RMM.t was more robust to censoring at various times than STM. Similar results are expected for RMM.p and GLMM compared to STM. Thus, the benefits of RMM.t, RMM.p, and GLMM observed here are conservative estimates because all individuals were censored at the same time.

### Genetic parameters

Estimates of genetic parameters for DGE and IGE from STM (Eq. 1) and RMM.t (Eq. 2) are in Table 1. Genetic parameters were expressed on the survival time scale (see Additional file 1), which demonstrates that it is possible to translate the variance components estimated with RMM.t to STM. Genetic parameters of survival time were very similar for the two models but prediction of breeding values was improved by using RMM.t compared to STM.

Estimates of genetic parameters for the same data were slightly different than those reported in Ellen et al. [15]. The total heritable variance relative to the phenotypic variance, *T*^2^, was estimated at 19 % in Ellen et al. [15], and 18 % in our study. In our study, the maximum number of survival days was cut off at 416 days, which was the maximum survival time of the youngest age group. In Ellen et al. [15], the maximum survival time was 447 days. Furthermore, Ellen et al. [15] included the average survival time of the back neighbours as a fixed covariate because hens shared drinking nipples. In our study, the fixed effect of survival of the back neighbours was excluded because this effect may indirectly result from the focal cage itself; if a back neighbour effect exists, then mortality in the focal cage will affect mortality of back neighbours, and vice versa, creating a feedback loop. Consequently, fitting a fixed effect for mortality of back neighbours might indirectly correct for the mortality observed in the focal cage itself, at least partly.

As in the study of Ellen et al. [15], covariances between direct and indirect genetic effects were positive for line W1 and negative for line WB. As described by these authors, with death due to cannibalism it is expected that the covariance between DGE and IGE will be negative because of strong competition. A positive covariance would mean that hens benefit from not harming others [12]. However, in our study, covariance estimates were not significantly different from zero.

## Conclusions

Our results indicate that including timing of IGE expression in analysis of survival reduces the accuracy of EBV for survival. Moreover, our results show that repeated measures models improve accuracy of EBV for survival time in laying hens. Although there was no universal best method, accuracies of EBV increased by up to 20 and 12 % for lines WB and W1, respectively. Thus, it is important to use methods that can incorporate censoring when analysing survival data, such as using a repeated measures model instead of a general linear mixed model to analyse survival data. This is an important finding since more accurate EBV contribute to increased rates of genetic gain.

## Additional files

10.1186/s12711-015-0152-2 Predicting phenotypes using STM, RMM.t, RMM.p, and GLMM. Detailed description on calculating predicted phenotypes using STM, RMM.t, RMM.p, and GLMM.
10.1186/s12711-015-0152-2 Translating breeding values and genetic parameters of RMM.t to survival days. Detailed description on translating breeding values and genetic parameters from RMM.t to survival days.
