# Supplementary material for: Predicting direct and indirect breeding values for survival time in laying hens using repeated measures
Source: Genet Sel Evol. 2015 Sep 28;47:75. doi: 10.1186/s12711-015-0152-2 (PMC4587788; doi:10.1186/s12711-015-0152-2)
Supplement: Supplementary file 1 — 10.1186/s12711-015-0152-2 Predicting phenotypes using STM, RMM.t, RMM.p, and GLMM. Detailed description on calculating predicted phenotypes using STM, RMM.t, RMM.p, and GLMM. [file 12711_2015_152_MOESM1_ESM.docx]

## Additional file 1 – Predicting phenotypes using STM, RMM.t, RMM.p, and GLMM

In this study, the quality of estimated breeding values was judged using cross validation for survival time. Known phenotypes were set to missing, their values were predicted, and finally the predicted values were correlated to the observed values. Predicted phenotypes were the rank of predicted survival times of individuals. Phenotypes were predicted by combining the estimated DGE ($\hat{A}_{D_{i}})$ of the individual itself and the estimated IGE of its cage mates (*n* = 3) that were present at the start of the experiment $(\sum\hat{A}_{I_{j}})$. Thus the predicted phenotype of individual *i* was

$\hat{P}_{i}= \hat{A}_{D_{i}}+\sum_{j=1}^{n-1} \hat{A}_{I_{j}}$,

where estimated breeding values $\hat{A}_{D_{i}}$ and $\sum_{j=1}^{n-1} \hat{A}_{I_{j}}$ refer to survival time. For STM, these estimated breeding values follow directly from the analysis, whereas for RMM.t, RMM.p, and the GLMM the estimated breeding values have to be translated to the survival time scale.

For RMM.t and RMM.p, the sum of all repeated measures of an individual *i* corresponds to the observed survival time in months of individual *i*. Hence, for RMM.t, predicted phenotypes were obtained as

$$\hat{P}_{i}=\sum_{t} \left( \left( \hat{A}_{D_{i}}+\sum_{j=1}^{n-1} \hat{A}_{I_{j}} \right)t \right),$$

and for RMM.p, predicted phenotypes were obtained as

$$\hat{P}_{i}=\sum_{t} \left( \left( \hat{A}_{D_{i}}+\sum_{j=1}^{n-1} \hat{A}_{I_{j}} \right)x_{t} \right),$$

with *x_t_* being a function of mean survival at time *t*, $x_{t}=\sqrt{p_{t}(1-p_{t})}$, *p_t_* denoting mean survival at time *t*.

The GLMM gives predictions on the underlying logit scale. Before calculating $\hat{P}_{i}$, these predictions need to be back transformed to the observed scale. Since $logit\left( E\left( y \right) \right)=log\left( p/\left( 1-p \right) \right)$, for any individual *i* at time *t,* the predicted survival probability is given by

$\hat{p}_{i,t}=\frac{e^{\mu(t)+\hat{A}_{D_{i}}+\sum_{j=1}^{n-1} \hat{A}_{I_{j}}}}{1+e^{\mu(t)+\hat{A}_{D_{i}}+\sum_{j=1}^{n-1} \hat{A}_{I_{j}}}}$,

where μ(t) represents the fixed effect solution at each period coming from $\eta\left( E\left( \mathbf{y} \right) \right)=\mathbf{Xb}$**,** being a sixth order polynomial of time. The fixed effect was included because the transformation from the underlying to the observed scale is non-linear. By including the mean, the DGE and IGE predictions are scaled to the mean population survival for each month. Finally, the predicted phenotype for the GLMM is the sum of the predicted survival probabilities at each point in time,

$\hat{P}_{i}=\sum_{t} \hat{p}_{i_{t}}$.
