# Supplementary material for: Predicting direct and indirect breeding values for survival time in laying hens using repeated measures
Source: Genet Sel Evol. 2015 Sep 28;47:75. doi: 10.1186/s12711-015-0152-2 (PMC4587788; doi:10.1186/s12711-015-0152-2)
Supplement: Supplementary file 2 — 10.1186/s12711-015-0152-2 Translating breeding values and genetic parameters of RMM.t to survival days. Detailed description on translating breeding values and genetic parameters from RMM.t to survival days. [file 12711_2015_152_MOESM2_ESM.docx]

## Additional file 2 – Translating breeding values and genetic parameters of RMM.t to survival days.

In this study, survival was modelled as being dead (0) or alive (1) each month. More interest is however in the number of survival days. This appendix shows how the breeding values, estimated genetic parameters, and phenotypic variance for survival can be translated to the survival time scale.

Survival time (*ST_i_*) of an individual *i* is the sum of its survival records (*S_i_*) at each day,

$${ST}_{i}=c\cdot\sum_{t_{1}}^{t_{2}} {S_{i}}_{t}$$

with *c* being a multiplication factor to translate monthly survival into days; *c* = 30.4, *t_1_* is the start of the laying period (in months), and *t_2_* the end of the laying period. The continuous time equivalent of this expression is

${ST}_{i}=c\cdot\int_{t_{1}}^{t_{2}} {S_{i}}_{t}dt$.

Substituting *ST_i_* by Equation 1 and $S_{i_{t}}$by Equation 2, ignoring the fixed effects, and using an animal model for survival time yields

$$A_{{ST,D}_{i}}+\sum_{j\neq i}^{n-1} A_{{ST,I}_{j}}+{cage}_{ST,k}+e_{ST,ijk}$$

$=c\int_{t_{1}}^{t_{2}} \left( A_{S,D_{i}}t_{l}+\sum_{j\neq i}^{n-1} A_{S,I_{j}}t_{l}+{cage}_{S_{kl}}+{cage}_{S_{k}}t_{l}+{{PE}_{S_{i}}t}_{l}+e_{S_{ijkl}} \right)dt$,

where subscript ST denotes survival time, and subscript S denotes survival. This expression identifies the following correspondence between the direct and indirect breeding values for survival time and survival,

$$A_{{ST,D}_{i}}=c\int_{t_{1}}^{t_{2}} {A_{S,D_{i}}t}_{l} dt$$

and

$\sum_{j\neq i}^{n-1} A_{{ST,I}_{j}}=c\int_{t_{1}}^{t_{2}} \sum_{j\neq i}^{n-1} A_{S,I_{j}}t_{l}dt$*.*

Solving the integrals shows how direct and indirect breeding values for survival are related to survival time,

$$A_{{ST,D}_{i}}=\frac{1}{2}c(t_{2}^{2}-t_{1}^{2})A_{S,D_{i}}$$

and

$A_{{ST,I}_{i}}=\frac{1}{2}c(t_{2}^{2}-t_{1}^{2})A_{S,I_{j}}$.

These two expressions can also be applied to the direct and indirect EBV. Taking the (co)variance shows that the relationship between the genetic parameters for survival time and survival is given by

.

These equations are used in Table 3 to translate estimated genetic parameters for survival to the survival time scale.

The relationship between the phenotype for survival time and the model for survival is given by (ignoring fixed effects)

$y_{ST,ijk}=$= $c\int_{t_{1}}^{t_{2}} \left( A_{S,D_{i}}t_{l}+\sum_{j\neq i}^{n-1} A_{S,I_{j}}t_{l}+{cage}_{S_{kl}}+{cage}_{S_{k}}t_{l}+{{PE}_{S_{i}}t}_{l}+e_{S_{ijkl}} \right)dt$,

Solving the integral yields

$y_{ST,ijk}=c\left( \frac{1}{2}\left( t_{2}^{2}-t_{1}^{2} \right)\left( A_{S,D_{i}}+\sum_{j\neq i}^{n-1} A_{S,I_{j}}+{cage}_{S_{k}}+{PE}_{S_{i}} \right)+\sum_{t1}^{t2} ({cage}_{S_{kl}}+e_{S_{ijkl}}) \right)$.

The last two terms remain summations because a different effect is fitted at each time point. Taking the variance on both sides shows that phenotypic variance for survival time is given by

$\sigma_{P,ST}^{2}=c^{2}\left[ \frac{1}{4} {{(t_{end}}^{2}-{t_{start}}^{2})}^{2} \left( \sigma_{A_{D}}^{2}+\left( n-1 \right)\sigma_{A_{I}}^{2}+\sigma_{{cage}_{k}}^{2}+\sigma_{PE}^{2} \right) \right.+\left. \left( t_{end}-t_{start} \right)\cdot\sigma_{{cage}_{km}}^{2}+\sum_{t=end}^{t=start} \sigma_{e}^{2}) \right]$.

The last term is kept as a sum because, in the survival model, a separate residual variance was fitted for each time point (whereas a variance common to all time points was fitted for the cage effect). This expression is used in Table 3 to calculate phenotypic variance on the survival time scale from the parameter estimates of the survival model.
